# Design and methods of the REMOVAL-HD study: a tRial Evaluating Mid cut-Off Value membrane clearance of Albumin and Light chains in HaemoDialysis patients

**DOI:** 10.1186/s12882-018-0883-8

**Published:** 2018-04-17

**Authors:** R. Krishnasamy, C. M. Hawley, M. J. Jardine, M. A. Roberts, Y. J. Cho, M. G. Wong, A. Heath, C. L. Nelson, S. Sen, P. F. Mount, E. M. Pascoe, D. Darssan, L. A. Vergara, P. A. Paul-Brent, N. D. Toussaint, D. W. Johnson, C. A. Hutchison

**Affiliations:** 1 Department of Nephrology, Sunshine Coast University Hospital, Birtinya, QLD Australia; 2 Centre for Kidney Disease Research, The University of Queensland, Brisbane, Australia; 3 Australasian Kidney Trials Network, The University of Queensland, Brisbane, Australia; 4 Department of Nephrology, Princess Alexandra Hospital, Brisbane, Australia; 5 The George Institute for Global Health, UNSW, Sydney, Australia; 6 San Renal Dialysis Unit, Sydney Adventist Hospital, Sydney, Australia; 7 Department of Nephrology, Concord Repatriation and General Hospital, Sydney, Australia; 8 Eastern Health Clinical School, Monash University, Melbourne, Australia; 9 Department of Nephrology, Western Health, Melbourne, Australia; 10 Department of Medicine, Western Health, University of Melbourne, Melbourne, Australia; 11 Western Health Chronic Disease Alliance, Western Centre for Health Research and Education, Western Health, St Albans, Australia; 12 Department of Nephrology, Austin Health, Melbourne, Australia; 13 Department of Nephrology, The Royal Melbourne Hospital, Parkville, Australia; 14 Department of Medicine, Hawke’s Bay District Health Board, Hawke’s Bay Hospital, Omahu Rd, Hastings, Hawkes Bay, New Zealand

**Keywords:** Albumin, Dialyser, Mid cut-off membrane, Efficacy, Free light chains, Haemodialysis, Safety

## Abstract

**Background:**

Removal of uraemic toxins is inadequate using current dialysis strategies. A new class of dialysis membranes have been developed that allow clearance of larger middle molecules. The REMOVAL-HD study (a tRial Evaluating Mid cut-Off Value membrane clearance of Albumin and Light chains in HaemoDialysis patients) will address safety, efficacy and the impact on patient-centred outcomes with the use of a mid cut-off (MCO) dialyser in a chronic haemodialysis (HD) population.

**Methods:**

REMOVAL-HD is an open label, prospective, non-randomised, single-arm, multi-centre device study in 85 chronic HD participants. All visits will be conducted during regular HD sessions and participants will undergo a 1 month wash-in period using a standardised high flux dialyser, 6 months of intervention with a MCO dialyser and 1 month of wash-out using a high flux dialyser. The primary endpoint is change in pre-dialysis concentrations of serum albumin, with secondary endpoints including the efficacy of clearance of free light chains and β-2 microglobulin, and patient-centred outcomes including quality of life, symptom burden, functional status, nutritional status, hospitalisation and death.

**Discussion:**

MCO dialysers are a novel form of HD membrane. The REMOVAL-HD study is a pivotal study designed to monitor the immediate and medium-term effects following exposure to this dialyser.

**Trial registration:**

Australian New Zealand Clinical Trials Registry Number (ANZCTRN) 12616000804482. Date of registration - 21/06/2016.

## Background

Haemodialysis (HD) remains a principal renal replacement modality for patients with end stage kidney disease (ESKD). Despite the efficacy of HD as a treatment to replace essential kidney functions, such as fluid and acid-base balance, the morbidity and mortality of patients receiving HD remain high when compared with those of the general population [1, 2]. The inadequate removal of uraemic toxins, particularly those in the middle molecule range (0.5–60 kDa), may play a role in this phenomenon [3]. Middle molecules are an important class of uraemic solutes which have been linked to reduced survival associated with ESKD [3, 4].

With the advent of high-flux dialysers and haemodiafiltration (HDF), the number of middle-molecules removed by chronic HD programs has continually increased but current HD processes principally only remove molecules with molecular weight cut-offs between 10 to 20 kDa [5]. There are still over 20 middle-molecules that are inadequately removed by current dialysis strategies but are potential contributors to chronic inflammation, cardiovascular disease, secondary immunodeficiency and reduced quality of life in dialysis patients [6, 7].

In recent years, there has been increasing interest in the development of new generations of dialysis membranes that will allow more effective removal of larger middle molecules. High cut-off HD membranes, with pore sizes of 8 to 10 nm, have been shown to improve clearance of larger solutes [8]. However, the use of high cut-off membranes was associated with substantial albumin loss and supplementation with human albumin solution was recommended at the end of each dialysis session [6]. These membranes were therefore deemed unsuitable in the setting of chronic HD.

A mid cut-off (MCO) dialyser (such as Theranova) has a pore size and molecular weight cut-off intermediate between those of either a high flux or a high cut-off membrane. MCO membranes are designed to provide increased clearance of larger middle-molecules in chronic HD patients, compared with high flux HD. Short term clinical studies following 4 dialysis sessions have demonstrated effective removal of molecules up to the molecular weight (MW) of 45 kDa [9]. However, these studies also identified a greater loss of albumin (MW 66.5 kDa) compared to high-flux dialysis and HDF [9]. It is currently unknown if this degree of albumin loss is transient or will be tolerated in chronic dialysis patients. Sustained albumin loss is a concern for a chronic dialysis treatment as hypoalbuminemia is strongly associated with increased morbidity and mortality in patients receiving HD [10]. In addition, to date clinical outcomes have not yet been studied with the sustained use of a MCO dialyser.

Thus, the purpose of the REMOVAL-HD study is to determine safety and efficacy of the MCO dialyser (Theranova; Baxter Healthcare, Sydney, Australia) in a chronic HD population over 6 months. This study will assess the efficacy of MCO dialysis regarding the clearance of larger middle-molecules, and determine its safety with regards to its effect on serum albumin.

## Methods/design

### Study aims

The primary objective of the REMOVAL-HD study is to determine the change in pre-dialysis concentrations of serum albumin in participants undergoing chronic HD using the MCO Theranova dialyser between baseline and 6 months. This study will also examine the trend of serum albumin change over the 6-month treatment period and the proportion of participants with a drop in serum albumin of > 5% below their baseline value. The study will also assess the efficacy of clearance of the three middle molecules: lambda free light chains (lambda-FLC, MW 45 kDa), kappa free light chains (kappa-FLC, MW 22.5 kDa) and β-2 microglobulin (MW 11 kDa).

### Study design and setting

**Fig. 1:**
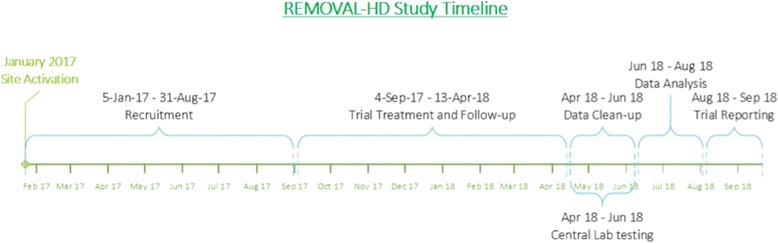
Overall study timeline

REMOVAL-HD is an investigator led, open label, non-randomised, single-arm, multi-centre device study. The study will involve 85 participants from 9 in-centre haemodialysis units in Australia and New Zealand. Table 1 summarizes the inclusion and exclusion criteria for the REMOVAL-HD study. Participants with urine output< 500 mL were included to minimise differences in clearance of measured molecules by residual renal function. The study is coordinated and supported by the Australasian Kidney Trials Network (AKTN) Fig. 1. demonstrates the overall study timeline. The study commenced in January 2017 and successfully completed recruitment in August 2017.

**Table 1:** Inclusion and exclusion criteria for the REMOVAL-HD study

Inclusion criteria
1. Established chronic in-centre haemodialysis (HD) patient (> 12 weeks on HD)
2. Aged over 18 years
3. Has a functioning Arteriovenous Fistula or Graft
4. Either oliguric (< 500 mL/24 h based on 24 h urine collection within 12 weeks of screening) or anuric
5. Able to give informed consent
Exclusion criteria
1. Planned renal transplant within study intervention period
2. Planned conversion to peritoneal dialysis or transfer to another dialysis unit within study intervention period
3. Active chronic infection or significant active inflammatory conditions including autoimmune disease, inflammatory arthritis and active malignancy
4. Life expectancy < 12 months
5. Pregnancy or breast feeding
6. Indication for haemodiafiltration (HDF) according to treating physician
7. Dialysis catheter in situ
8. Receiving immunosuppressant medication
9. Current use of nutritional or dietary supplements to increase or reduce protein intake including protein powder or weight loss supplements and is unable to cease the supplement
10. Serum albumin < 30 g/L (within 4 weeks of screening)
11. Inability to complete study assessments

### Ethical considerations

Ethical approval has been obtained from Institutional Ethics Committees (IEC) for each participating site (Protocol version 1.3 11th November 2016). The study will be performed in accordance with the 2013 Fortaleza, Brazil 7th Revision of the Declaration of Helsinki, the National Health and Medical Research Council (NHMRC) Statement on Ethical Conduct of Human Research (2015), Joint NHMRC/AVCC Statement and Guidelines on Research Practice (1997), applicable ICH guidelines, ISO 14155:2011 and Note for Guidance on Good Clinical Practice (CPMP/ICH/135/95) annotated with Therapeutic Goods Administration (TGA) comments. Informed signed consent will be obtained from all participants. This study is registered with Australian and New Zealand Clinical Trials Registry (ANZCTRN 12616000804482).

### Study procedures

**Fig. 2:**
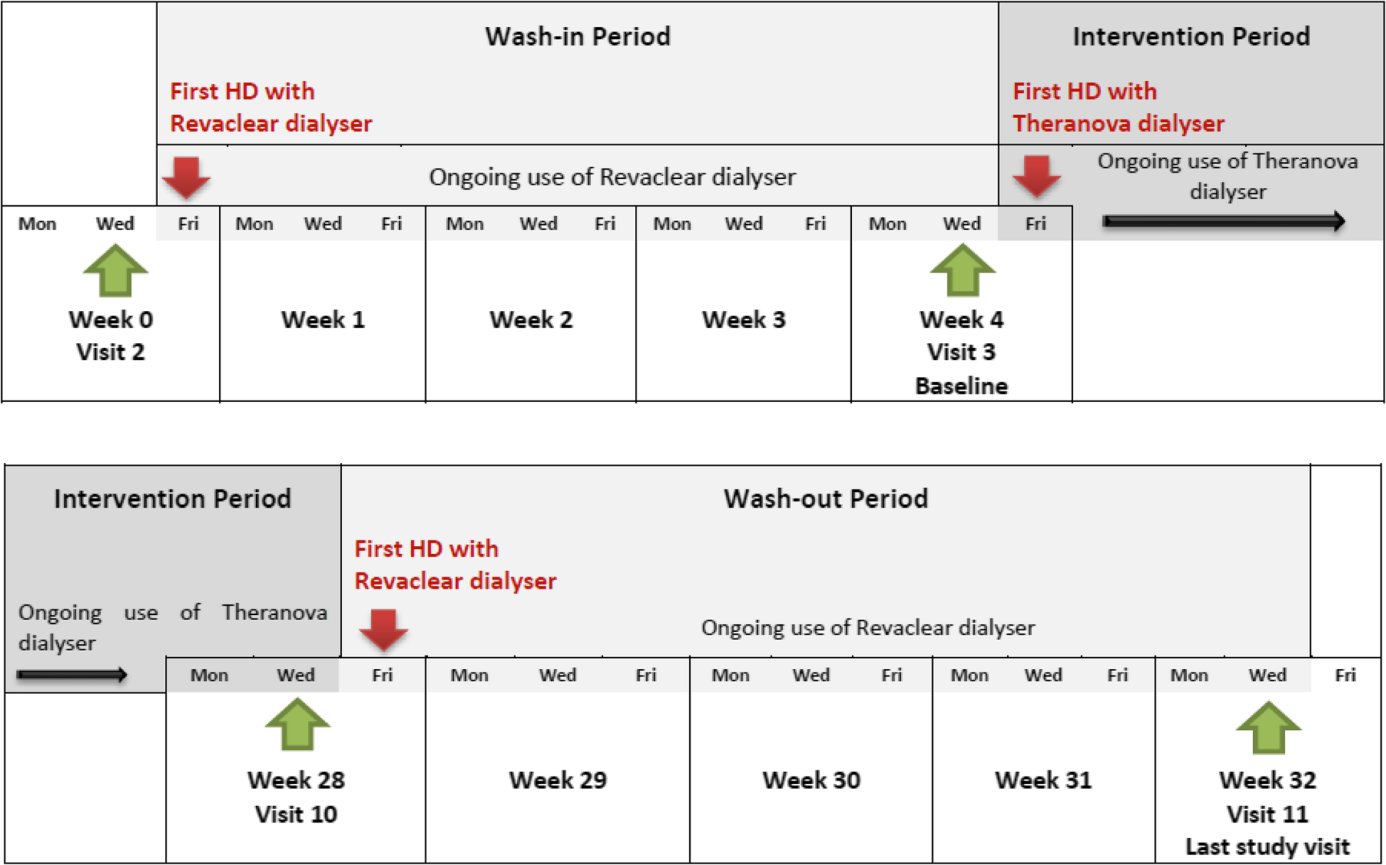
Timing of visits and changes in study treatment

The timing of the study visits and changes in study treatment are shown in Fig. 2. All visits will occur when participants attend their usual HD session. Participants will undergo a wash-in, intervention and wash-out period, as outlined below.

#### Wash-in period (week 0–4)

Enrolled participants will receive a 4-week wash-in period using a high-flux dialyser (Revaclear; Baxter Healthcare, Sydney, Australia).

#### Intervention period (week 4–28)

Participants will then receive 24 weeks of treatment with a MCO dialyser (Theranova; Baxter Healthcare, Sydney, Australia) three times per week.

Although dialysis prescriptions will remain under the supervision of the local nephrology team, the following treatment guidance is provided:

- target blood flow > 300 mL/min;
- dialysate flow rate 500 mL/min;
- dialysis session length and frequency to remain unchanged; and
- fluid removal according to participant’s individual prescription.

#### Wash-out period (week 28–32)

Participants will then receive a 4-week wash-out period using the Revaclear high flux dialyser.

### Data collection and outcome measures

The study visit schedule and timing of data collection are summarized in Table 2. Demographic and medical history, including age, gender, height and weight, race, blood pressure, heart rate, cause of ESKD, comorbidities, dialysis and medication history will be collected. Blood samples will be taken pre-dialysis at the mid-week HD session for local and central laboratory testing. Central samples will be centrifuged and stored at − 80 °C until analysed.

**Table 2:** Visit schedule for REMOVAL-HD study

Study Phase	Screening	Wash in Period		Intervention							Wash out
Visits	Visit 1	Visit 2	Visit 3 Baseline	Visit 4	Visit 5	Visit 6	Visit 7	Visit 8	Visit 9	Visit 10	Visit 11
	Week −1	Wk 0	Wk 4	Wk 6	Wk 8	Wk 12	Wk 16	Wk 20	Wk 24	Wk 28	Wk 32
Screening											
Inclusion/exclusion criteria	X	X									
Informed Consent	X										
24 h Urine collection	X										
Demographics/Medical History/Physical exam		X									
Primary Outcome											
Centrally tested serum albumin^a^		X	X	X	X	X	X	X	X	X	X
Trial Intervention Use											
High Flux (Revaclear) dialyser		X	X							X	X
Mid cut-off (Theranova) dialyser			X	X	X	X	X	X	X	X	
Clinical assessments											
Erythropoietin Resistance Index			X				X			X	
Weight (pre & post HD)			X				X			X	X
Duration of HD			X				X			X	X
Restless Leg Symptom Rating Scale			X				X			X	
Malnutrition Inflammation Score			X				X			X	
Edmonton Symptom Assessment System Revised			X				X			X	
6 min walk test^a^			X				X			X	
Adverse events (as required)		X	X	X	X	X	X	X	X	X	X
Local lab assessments											
Albumin^a^		X	X	X	X	X	X	X	X	X	X
Urea (pre & post HD)			X				X			X	
Haemoglobin, transferrin, INR, APTT^a^			X				X			X	
Central lab samples											
Lambda free light chains^a^		X	X	X	X	X	X	X	X	X	X
Κappa-FLC, β2Microglobulin, high sensitivity C-Reactive Protein^a^			X				X			X	
Substudy – Matrix Gla Protein, fetuin A, CPP, FGF23^a^			X				X			X	

^a^ Collected pre-dialysis prior to the mid-week HD

*APTT* activated partial thromboplastin time, *CPP* calciprotein particles, *FGF23* Fibroblast growth factor23, *HD* haemodialysis, *INR* international normalised ratio

#### Primary outcome measure

The primary outcome is change in pre-dialysis serum albumin between baseline and at 6 months. The study will also monitor the trend of changes in pre-dialysis concentrations of serum albumin during the intervention period. Serum albumin levels for these analyses will be obtained from central laboratory testing following completion of all study visits. Serum albumin will also be monitored at every visit locally to record safety data, including any large reduction (> 25%) in serum albumin level.

#### Secondary outcome measures

1. Change in centrally tested middle molecules, including lambda-FLC, kappa-FLC and β-2 microglobulin.
2. Change in inflammatory marker (high sensitivity C-Reactive Protein) and coagulation profile [international normalised ratio (INR) / (activated partial thromboplastin time (APTT)].
3. Erythropoietin resistance index (ERI) in participants taking erythropoietin (EPO) (ERI ≥1.0 IU/kg/week/gHb) or darbepoetin (DPO) (ERI ≥0.005 μg/kg/week/gHb).
4. Assessment of dialysis related symptoms:
  - Restless leg syndrome [Restless Legs Syndrome Rating Scale (RLSRS)].
  - Quality of life [Edmonton Symptom Assessment System Revised (ESAS-R)] [11].
5. Functional status with 6-min walk test – the distance a participant can achieve by walking on a flat surface in 6 min is measured using trundle wheel.
6. Nutritional status using Malnutrition Inflammation Score (MIS) [12].
7. Number and duration of all-cause hospitalisations.
8. Number of infection-related hospitalisations.
9. All-cause mortality.

#### Exploratory outcome measures

The impact on circulating levels of calcification regulatory proteins, including matrix Gla protein (MGP), fetuin-A, calciprotein particles (CPPs) and fibroblast growth factor 23 (FGF23) will be explored.

#### Frequency of measurement of biomarkers

Serum albumin and lambda FLC measurement will be evaluated at every study visit. However, the remaining assessments will be performed at baseline, midway through the study and at completion of the intervention period (Table 2).

### Sample size estimation

The study has been powered to identify a change of 5% in serum albumin concentrations from a median baseline level of 35 g/L (standard deviation of 5 g/L). A 5% decline in serum albumin has been shown to be associated with a doubling of mortality risk in haemodialysis patients [13]. To detect this change with 80% power at the 5% significance level, a sample size of 72 is needed. Allowing for a 15% loss to follow-up, a minimum of 85 participants need to be recruited to achieve the required sample size.

### Statistical analysis approach

The 95% confidence interval for the mean difference in central serum albumin between 6 months and baseline will be used to assess the change in albumin: a clinically non-meaningful reduction in albumin due to MCO dialysis will be inferred if the lower limit of the confidence interval excludes a reduction in serum albumin that is equal to or exceeds 5% of the median serum albumin at baseline. Change in serum albumin levels over the entire intervention period (weeks 4 to 28) will be analysed using a linear mixed-effects regression model. This analysis will be adjusted for baseline serum albumin levels. Other continuous secondary outcome variables will be analysed using the same methods described above. Secondary outcomes that are categorical will be analysed using McNemar’s test for comparisons between 6 months and baseline and generalised estimating equations for trends across the intervention period. Serious adverse events (SAE), serious adverse device effects (SADE) and unanticipated serious adverse device effects (USADE) will be presented as number (percent) to assess the safety of the MCO dialyser. All hypothesis tests will be assessed at 5% level of significance.

### Safety reporting

For the purposes of this study, the period of observation for collection of treatment-related SAEs will be from the start of the wash-in period until the end of the wash-out period. All SAEs will be recorded regardless of whether they are related to the study intervention. SADEs, USADEs and SAEs will be recorded as part of the regular data collection activities of the trial.

### Data monitoring

Safety will be examined through close monitoring of data from individual participants rather than through statistical comparison. This will be achieved by continuous monitoring of serum albumin for any large reductions from baseline (> 25%) in serum albumin level. It will be the responsibility of the Trial Steering Committee (TSC) to protect the safety of trial participants and the scientific integrity of the trial by overseeing the monitoring of albumin levels, SAEs and operational data. For the purpose of data validation, the principal investigators will permit a member of the AKTN or its designee to inspect the source data and compare them with the case report forms. Notification of these audits will be sent to all investigators in advance.

### Discontinuation of study invention

Participants will revert to high-flux HD/HDF at any stage if any of the following occur:

- an unexplained reduction in pre-dialysis serum albumin of > 25% at 2 consecutive treatment visits compared to baseline level. Potential explanations for reductions in albumin levels may include infections and inflammatory conditions, such as autoimmune disease, inflammatory arthritis and malignancy;
- failure to maintain adequate HD dose measured according to local guidelines;
- at the discretion of the treating physician.

When withdrawn from the study intervention, participants will continue to be followed up as per the trial procedures.

## Discussion

Patients with ESKD on HD have a disproportionally heightened risk of mortality when compared to the general population. Retention of uraemic toxins may be a key driver of accentuated mortality in HD patients through multifaceted mechanisms, such as promotion of inflammation, endothelial dysfunction and fibrosis [14]. However, therapies targeting improved clearance of uraemic toxins, especially middle molecules, are limited and only partially effective. Kirsch et al. recently demonstrated that MCO dialysers resulted in greater clearance of larger middle molecules compared to standard high flux HD and HDF in 39 participants over 4 dialysis treatment sessions [9]. However, albumin losses were greater in the MCO group (median levels from 2.9 to 7.3 g), although the long-term tolerability of this dialyser and the effect on serum albumin were unknown. Thus, REMOVAL-HD is a pivotal study that will determine if regular HD using MCO dialyser in a chronic HD population is safe and specifically will not result in a significant loss of albumin. The study will also assess the efficacy of removal of larger middle molecules using this dialyser.

### Rationale for single-arm design

The MCO dialyser is a novel form of HD therapy. There is insufficient evidence regarding the safety and efficacy of this treatment in HD patients. The immediate and long-term effect following exposure to the dialyser will be examined in this single-arm study design. The results will inform the design of future, large-scale randomised trials using the MCO dialysers.

### Justification for selecting change in serum albumin as the primary outcome measure

Serum albumin concentration is widely regarded as a surrogate for “health and a good prognosis” in the dialysis population. Serum albumin is strongly associated with survival [15]. Serum albumin concentration has anti-oxidant properties and its concentration represents both a patient’s nutritional status and inflammatory burden [16, 17]. Therefore, any new treatment which can potentially reduce serum albumin concentrations, such as with the MCO dialyser, must be considered with care.

### Justification for selecting lambda FLC for assessment of middle molecules clearance

There are two circulating isotypes of free light chain - kappa monomer (22.5 kDa) and lambda dimer (45 kDa) - that are metabolized and cleared by the kidneys. Thus, FLC concentration increases with progressive decline in renal function. There is emerging evidence to support the notion that higher FLCs may drive uraemic inflammation, endothelial dysfunction and ultimately poorer survival in this cohort [18, 19]. In addition, the assays used to measure lambda FLC are well validated and have been used commercially across a wide range of platforms [20]. Of the two FLC isotypes, the higher molecular weight of lambda provides the greatest discriminatory value for comparing the increased clearance of middle molecules offered by Theranova compared with conventional high-flux membranes.

### Choice of secondary clinical measures

After primary safety and efficacy assessment, this study will provide exploratory measures of the potential for increased removal of larger middle molecules to provide patients with clinical benefit. The measures chosen: restless leg syndrome; six-minute walk test (functional status); malnutrition inflammation score (nutritional status); and quality of life, all have validated tools for measurement and are linked to the retention of larger middle molecules. In addition to these variables, the study will document the clinical end points of hospitalisation (total and infection related) and mortality.

## Conclusion

The MCO dialyser represents a new class of dialysis membrane with a greater ability to remove the majority of circulating middle molecules. REMOVAL-HD is a pivotal, open label, non-randomised, single-arm, multi-centre device study designed to provide novel insights into medium–term safety and efficacy of MCO dialyser.

## Abbreviations

AKTN: Australasian Kidney Trials Network
ANZCTRN: Australian New Zealand Clinical Trials Registry Number
APTT: Activated partial thromboplastin time
CPPs: Calciprotein particles
DPO: Darbepoetin
EPO: Erythropoietin
ERI: Erythropoietin resistance index
ESAS-R: Edmonton symptom assessment system revised
ESKD: End stage kidney disease
FGF23: Fibroblast growth factor 23
HD: Haemodialysis
HDF: Haemodiafiltration
IEC: Institutional ethics committee
INR: International normalised ratio
kappa-FLC: Kappa free light chains
kDA: kiloDalton
lambda-FLC: Lambda free light chains
MCO: Mid cut- off
MGP: Matrix Gla protein
MIS: Malnutrition inflammation score
NHMRC: National Health and Medical Research Council
nm: Nanometer
REMOVAL-HD: A trial evaluating mid cut-off value membrane clearance of albumin and light chains in haemodialysis patients
RLSRS: Restless legs syndrome rating scale
SADE: Serious adverse device effect
SAE: Serious adverse event
TGA: Therapeutic goods administration
TSC: Trial steering committee
USADE: Unanticipated serious adverse device effect
